# Development of a deep learning model for automated detection of calcium pyrophosphate deposition in hand radiographs

**DOI:** 10.3389/fmed.2024.1431333

**Published:** 2024-10-23

**Authors:** Thomas Hügle, Elisabeth Rosoux, Guillaume Fahrni, Deborah Markham, Tobias Manigold, Fabio Becce

**Affiliations:** ^1^Department of Rheumatology, University Hospital Lausanne (CHUV), University of Lausanne, Lausanne, Switzerland; ^2^Department of Diagnostic and Interventional Radiology, Lausanne University Hospital (CHUV) and University of Lausanne (UNIL), Lausanne, Switzerland; ^3^Department of Rheumatology, Inselspital, University Hospital Bern and University of Bern, Bern, Switzerland

**Keywords:** CPPD, chondrocalcinosis, machine learning, radiograph (X-ray), detection, image recoginiton, automated

## Abstract

**Background:**

Calcium pyrophosphate deposition (CPPD) disease is a leading cause of arthritis, which can mimic or strongly interfere with other rheumatic diseases such as gout, osteoarthritis (OA) or rheumatoid arthritis (RA). In the recently established ACR/EULAR CPPD classification criteria, calcification and OA features of the wrist and hand joints are substantial features.

**Objectives:**

To develop and test a deep-learning algorithm for automatically and reliably detecting CPPD features in hand radiographs, focusing on calcification of the triangular fibrocartilage complex (TFCC) and metacarpophalangeal (MCP)-2 and -3 joints, in separate or combined models.

**Methods:**

Two radiologists independently labeled a dataset of 926 hand radiographs, yielding 319 CPPD positive and 607 CPPD negative cases across the three sites of interest after adjudicating discrepant cases. CPPD presence was then predicted using a convolutional neural network. We tested seven CPPD models, each with a different combination of sites out of TFCC, MCP-2 and MCP-3. The model performance was assessed using the area under the receiver operating characteristic (AUROC) and area under the precision-recall (AUPR) curves, with heatmaps (Grad-CAM) aiding in case discrimination.

**Results:**

All models trialed gave good class separation, with the combined TFCC, MCP-2 and MCP-3 model showing the most robust performance with a mean AUROC of 0.86, mean AUPR of 0.77, sensitivity of 0.77, specificity of 0.80, and precision of 0.67. The TFCC-alone model had a slightly lower mean AUROC of 0.85 with a mean AUPR of 0.73. The MCP-2-alone and MCP-3-alone models exhibited mean AUROCs of 0.78–0.87, but lower mean AUPRs of 0.29–0.47. Heatmap analysis revealed activation in the regions of interest for positive cases (true and false positives), but unexpected highlights were encountered possibly due to correlated features in different hand regions.

**Conclusion:**

A combined deep-learning model detecting CPPD at the TFCC and MCP-2/3 joints in hand radiographs provides the highest diagnostic performance. The algorithm could be used to screen larger OA or RA databases or electronic medical records for CPPD cases. Future work includes dataset expansion and validation with external datasets.

## Introduction

Calcium pyrophosphate deposition (CPPD) disease encompasses a range of conditions, including calcium pyrophosphate (CPP) crystal arthritis (acute and chronic forms) and osteoarthritis (OA) (1). This disease affects hyaline and fibrocartilage such as the meniscus, visible as “chondrocalcinosis” (CC) on radiography, computed tomography, or ultrasound imaging.

The release of CPP crystals into the synovial fluid may result in microcrystalline arthritis, which can resemble or coincide with other arthritic conditions such as gout, RA, or rapidly progressive OA. The European Alliance of Associations for Rheumatology (EULAR) and the American College of Rheumatology (ACR) have recently published classification criteria for CPPD disease (2). A key feature of CPPD in hand radiographs is the presence of calcifications within the triangular fibrocartilage complex (TFCC) (or lunotriquetral ligament) or finger joints. Additional diagnostic criteria include joint space narrowing in different hand joints, especially the metacarpophalangeal (MCP) joints of the index and middle fingers (MCP-2 and MCP-3), and scaphotrapeziotrapezoidal (STT) joint.

The incorporation of automated image recognition technology marks a significant milestone, extending its application into the field of rheumatology (3). The majority of the so far FDA-approved AI algorithms are in the field of image recognition (4). The success of AI models in radiology is attributed to the use of radiographs as static, labeled datasets for input, and the execution of clinically meaningful classification tasks as output. Convolutional neural networks (CNNs) have proven to be a robust technology for image recognition, capable of classifying radiological images either standalone or in conjunction with clinical data to forecast disease progression (5). In CPPD, imaging remains a diagnostic hallmark, especially in the absence of laboratory evidence of CPP crystals in the synovial fluid. Ultrasound has developed into an efficient bedside tool, but radiography is still important, e.g., to determine the degree of OA or to rule out other pathologies (6).

The objective of this study was to develop and test a predictive deep-learning model for CPPD using hand radiographs and a labeled dataset that indicates the presence of CPPD at specific sites of interest (TFCC, MCP-2, MCP-3). As a rationale, the novel ACR/EULAR CPPD classification criteria permit the classification of CPPD disease based on clinical and radiological signs, without the need to identify CPP crystals or synovitis (2, 7, 8). Hence, algorithms designed to evaluate radiological features of CPPD could serve as valuable research tools, especially in larger datasets such as clinical registries. They would also facilitate the detection and scoring of CPPD features in datasets with concomitant OA or RA and could thus explore the role of CPPD as a largely ignored factor in these disorders. In this work, we primarily explored the feasibility and interpretability of various deep-learning models for detecting CPPD features in hand radiographs.

## Methods

### Dataset

Ethical approval from the local committee was obtained for this study (CER-VD protocol 2020–00033). The dataset consisted of DICOM files with 12-bit pixel data, containing radiographs with a single posteroanterior (PA) view of both hands with a few containing a single PA view of one hand. In total, we labeled a dataset of 926 hand radiographs, yielding 319 CPPD positive and 607 CPPD negative radiographs. The mean age of the patients was 64.5 years, 63% were females.

#### Labeling

Hand radiographs were assessed by two independent board-certified radiologists, with adjudication by a third senior musculoskeletal radiologist (with 16 years of experience) in case of disagreement, and binary labels (CPPD present or not) provided for the three sites of interest (TFCC, MCP-2, MCP-3). CMC1 and STT joints were omitted. The number of positive and negative cases per site and overall (a hand was considered positive overall if at least one site is classified as positive) are summarized in Table 1. Age and sex distribution per CPPD site are reported in Supplementary material S1. Interobserver agreement per CPPD site is reported in Supplementary material S2.

**Table 1:** Information for the class distribution and mean AUROC and AUPR results for the single site and combined models using five-fold cross-validation.

Model	Positive	Negative	AUROC	AUPR
TFCC, MCP2, MCP3	319	607	0.86+/−0.02	0.77+/−0.04
TFCC	282	644	0.85+/−0.02	0.73+/−0.02
MCP2	73	853	0.78+/−0.05	0.29+/−0.09
MCP3	91	835	0.77+/−0.04	0.47+/−0.08

The standard deviation of the AUROC and AUPR in each case is also provided.

#### Preprocessing

The pre-processing script was written in Python 3.11, predominantly making use of the scikit-image (v0.21.0) package and performed the following steps:

#### Hand separation

**Figure 1:**
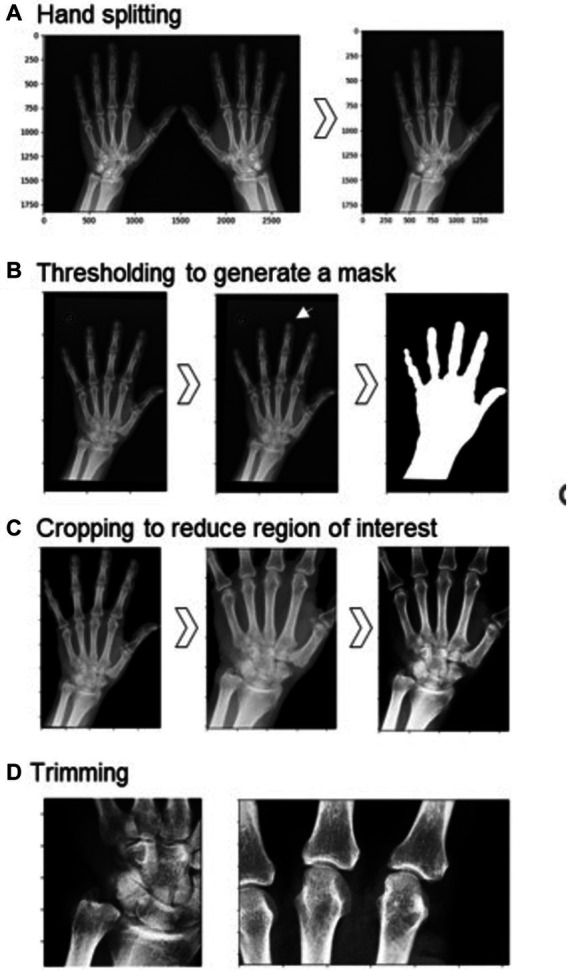
Preprocessing steps before training the model. (A) Hand splitting. (B) Thresholding to generate a mask of the hand region (original image on left, mask on the right). Other features were sometimes retained, as in the case of the circled ‘G’ (center), thus smaller objects needed to be removed (right). (C) Cropping reduced the region of interest to part of the hand containing the TFCC, MCP-2, and MCP-3, and contrast enhancement was performed on this reduced region. (D) Further trimming to two smaller regions: on the left, the TFCC region; on the right, the MCP-2 and MCP-3 joints.

Images containing the left and right hands were split into two separate images to be processed and analyzed separately (Figure 1). This was done by identifying the minimum of the mean pixel intensity in a vertical slice of the middle third of the pixel data. The right hand underwent vertical mirroring such that all images had the same hand orientation.

#### Segmentation

The border of the digital radiograph was identified and a binary erosion performed to eliminate image artifacts on the edges. A mask of the hand was created by first smoothing the image using a Gaussian function with a sigma of 3, and then using the otsu threshold to binarize the image (Figure 1B). Following this, we cropped the image to the hand region, retaining bones and the majority of the soft tissue. Occasionally, other regions of noise or labels were present, thus we removed all smaller remaining objects after thresholding to retain only the hand region. The MCP joint for digits 2 and 3, and the TFCC region are the areas used for the human classification of this dataset as CPPD positive or negative. Therefore, after filling in any holes in the hand region, we trimmed our images on each side (30% from the top, 5% from the base, 10% from the left, 20% from the right) to reduce our region of interest. Finally, we enhanced the contrast by using contrast-limited adaptive histogram equalization and adjusting the gamma contrast using a gamma of 1.5 and a gain of 1. Images were rescaled by converting the pixel values to a float between 0 and 255 and resized to a square with sides of 224 pixels. To develop site-specific models, we create two more zoomed-in regions: (i) for TFCC predictions; and (ii) for MCP-2 and MCP-3 predictions.

#### Model development and evaluation

The models were built, trained and evaluated using Keras (v2.13.1). We usedEfficientNetB4 as a base model (9) and took advantage of transfer learning: initially we used weights obtained from the ImageNet database for our base EfficientNetB4 model, and only trained on the additional layers specific to our model: (i) global average pooling; (ii) dense layer with an output of 16; and (iii) dense layer with an output of 1. Following this, we fine-tuned the model by unfreezing all layers and retraining. As our dataset was imbalanced, we used class weightings inversely proportional to their respective frequencies to reduce bias toward the negative class. For model training, we used the Adam optimizer with a learning rate of 1e-3 over 10 epochs for the transfer learning step, and a learning rate of 1e-4 over 8 epochs for fine-tuning, evaluating the loss using the binary cross-entropy loss function. From the three sites of interest (TFCC, MCP-2, MCP-3) there are seven possible models. For model comparison, we compared these models on identical input images (using the region showing all three sites of interest, as in the image on the far right of Figure 1C) using 80% of the data for training and 20% for testing, stratified on the MCP-3 dataset. Additionally, we investigated the combined, TFCC, MCP-2 and MCP-3 models alone using stratified five-fold cross-validation to obtain the mean AUROC and AUPR for each of these alternatives. In this case, the input images for the combined model are the same as before (far right of Figure 1C) while the input images for the single-site models are further cropped, as seen in Figure 1D. Finally, we examined a specific threshold on a fold of the combined model providing a confusion matrix, along with sensitivity, specificity and precision metrics.

#### Interpretability

To understand the decision-making taking place in our model, we applied the Grad-CAM technique to our last convolutional layer (“top_conv” in our base model, EfficientNetB4).

## Results

### Algorithm performance

**Figure 2:**
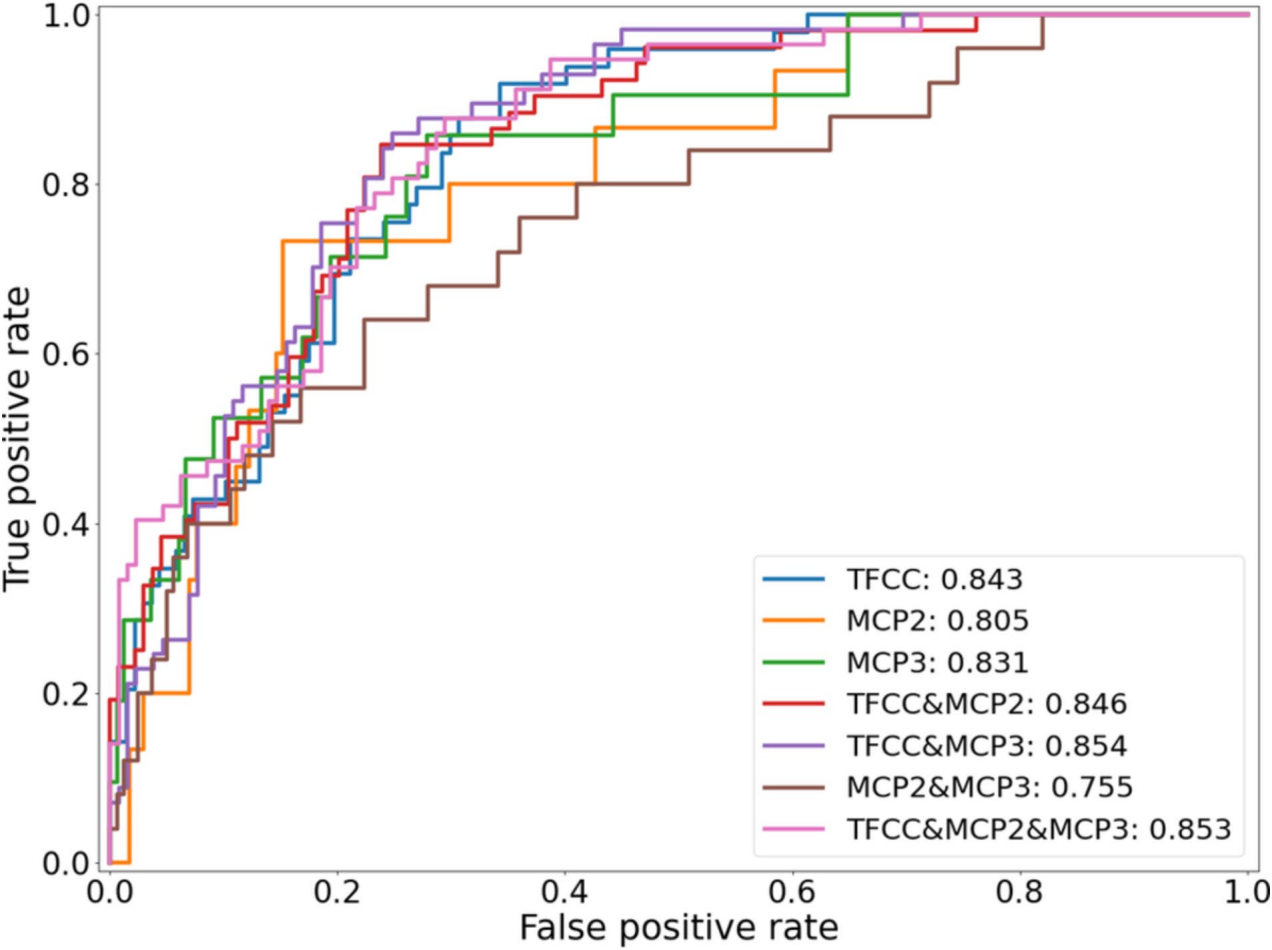
ROC curves for each of the seven different potential CPPD models, using an identical training and testing dataset.

Figure 2 shows the ROC curves for the seven potential CPPD models. All models showed predictive ability, with clear class separation. The combined TFCC, MCP-2 and MCP-3 model showed the highest performance with an AUROC of 0.85. The TFCC model performed the best out of any single-site model (AUROC of 0.84, compared to 0.81 and 0.83 for the MCP-2 and MCP-3 models, respectively). Combining information from the different sites increased the AUROC when TFCC was included, however decreased the AUROC if only the MCP sites were included: a model based on MCP-2 and MCP-3 gave the lowest AUROC of 0.76.

**Figure 3:**
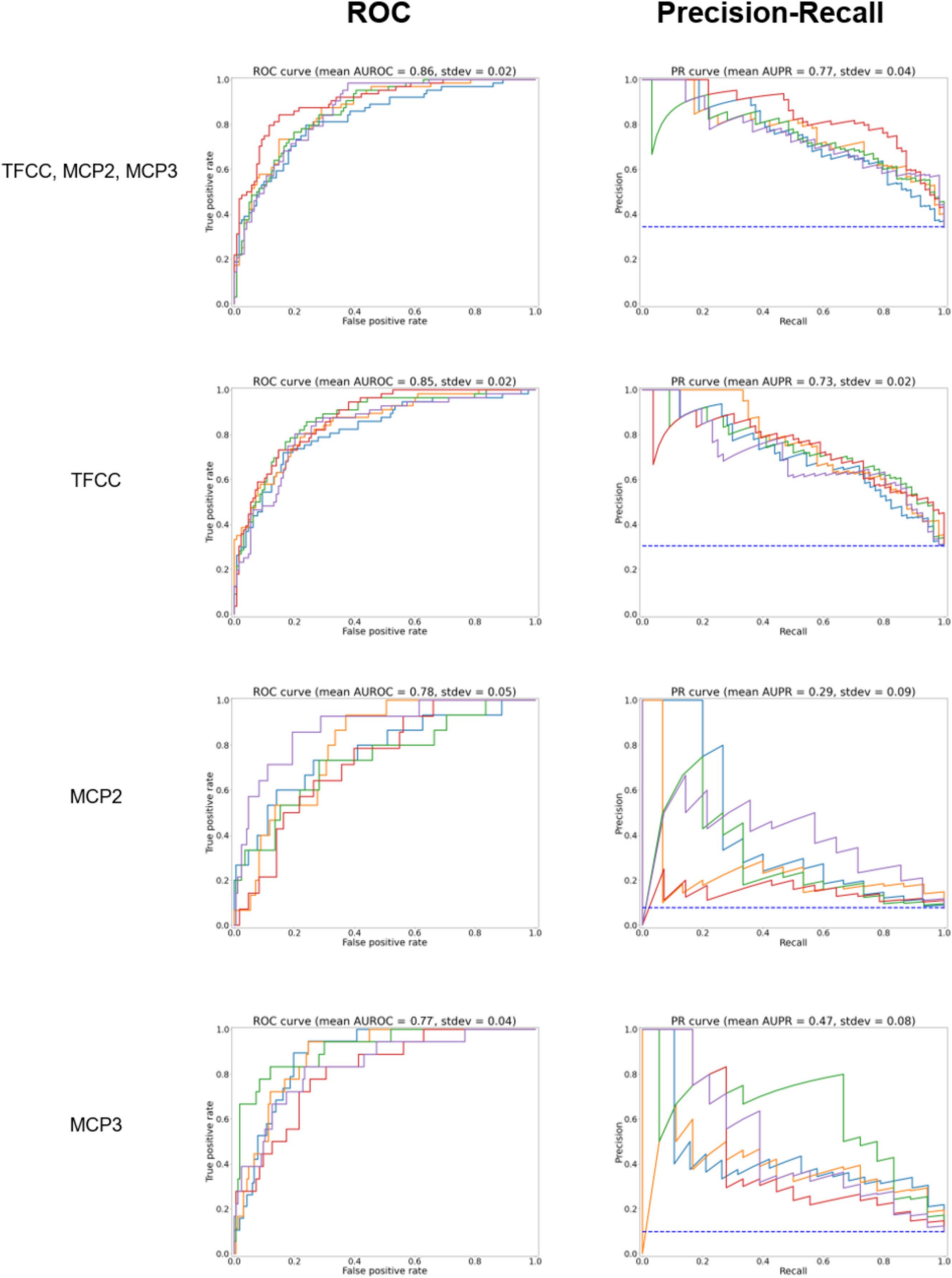
ROC curves for the model predicting whether any site (TFCC, MCP-2, MCP-3), TFCC alone, or MCP2 or MCP3 alone are positive for CPPD after further trimming of the image (test set, according to Figure 1D). On the right, precision to recall curves are shown. Different colors correspond to five-fold cross-validation.

**Figure 4:**
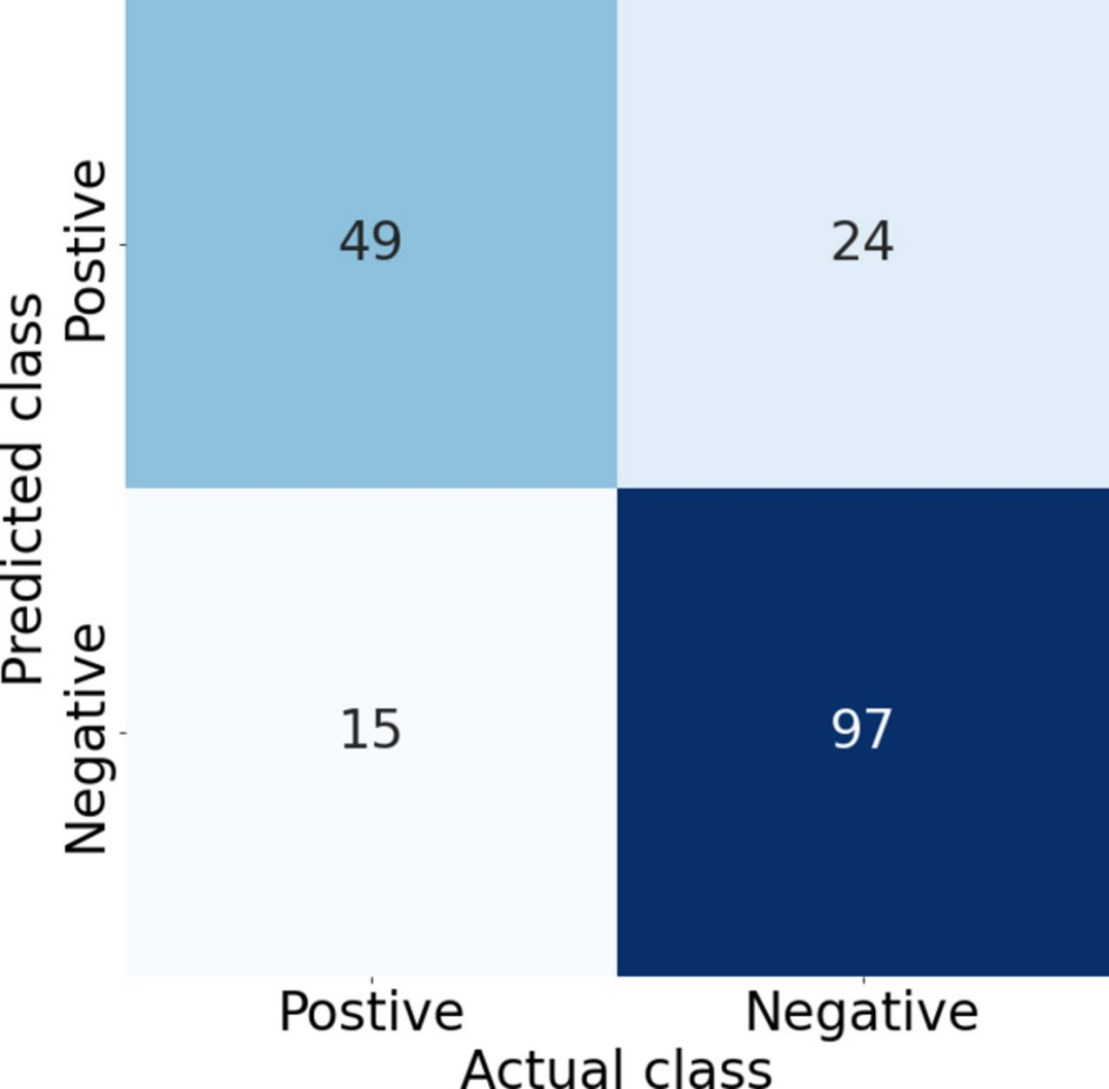
Confusion matrix corresponding to a threshold of 0.7 on one of the test folds of the combined model, giving a sensitivity (recall) of 0.77, specificity of 0.80, precision of 0.67, and F1 score of 0.72. Increasing the threshold increases the specificity and precision but decreases the sensitivity (recall), thus the threshold needs to be selected based on the clinical requirements.

The results of the five-fold cross-validation for the full combined and single-site models can be seen in Table 1. The ROC and PR curves for these models are seen in Figure 3. Again, we found good class separation for all models with the highest performance seen in the combined and TFCC models. The MCP-2 and MCP-3 models in this case, where the input images have been trimmed to a smaller region of interest, showed lower performance than in Figure 2 where the input images included the TFCC region. The confusion matrix using a threshold of 0.7 on a single fold of the combined model can be seen in Figure 4. This corresponds to a sensitivity (recall) of 0.77, a specificity of 0.80, and a precision of 0.67.

#### Interpretability

**Figure 5:**
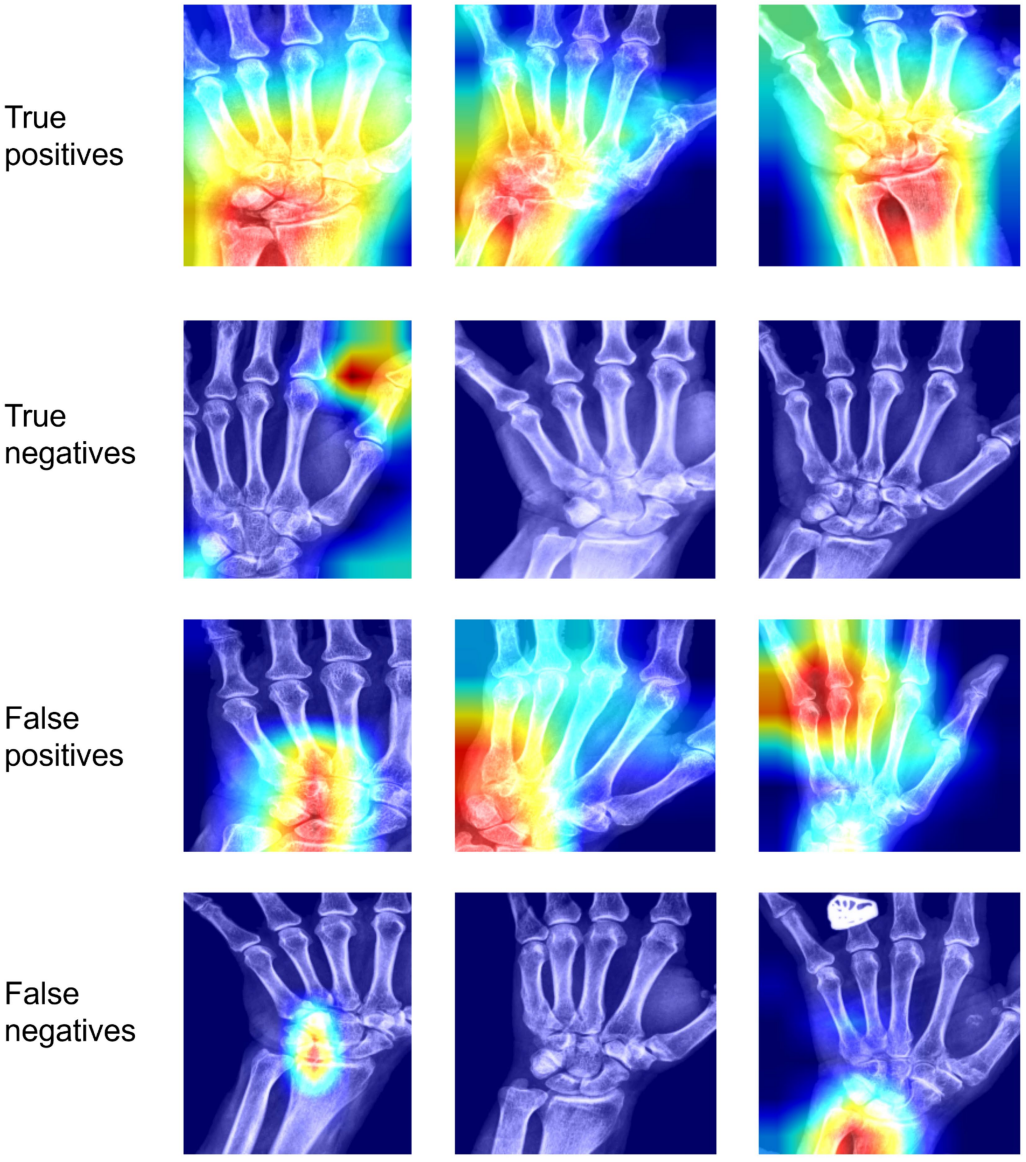
Interpretability results using Grad-CAM on the last convolutional layer for classifying any site (out of TFCC, MCP-2, MCP-3) as positive. In the first row, we see examples of images classified correctly as positive; in the second row, images classified incorrectly as positive; in the third row, images classified correctly as negative; in the fourth row, images classified incorrectly as negative.TP, True positive; FP, false positive; TN, true negative; FN, false negative.

To understand the decision-making taking place in our model, we applied the Grad-CAM (10) technique to the last convolutional layer in our base model. Example heatmaps can be found in Figure 5 for the model classifying positive cases as those where any site (out of TFCC, MCP-2, MCP-3) is positive. In general, cases classified as positive (true positive and false positives) have a large amount of activation focused on the hand region. In some true positive images the activated area is mostly focused on the regions of the hand labeled as positive. However, in some true positive images the highlighted regions are not those we anticipated, due to correlated features in other areas of the hand. These may be correlated due to features correlated with the presence of calcifications, or they may be features due to other correlated conditions. Similarly, false positives show activation in regions of interest, but also in other regions of the hand. Negative cases (both positive and negative), show very little activation. Where present, it tends to be in the background or in regions of the hand not being considered for the labels in our model. The model seems to focus less on MCP joints as compared to the wrist.

## Discussion

In this study, we present the initial steps of a deep-learning model that recognizes CPPD in hand radiographs at different sites. Leveraging the latest ACR/EULAR CPPD classification criteria, our findings underscore the advantage of employing a composite model that predicts a combination of radiographic CPPD features. The model demonstrates robust class differentiation, both in the combined classification and the TFCC-specific analysis. Using MCP lesions alone is less reliable for detecting CPPD, possibly due to a limited number of positive instances in the dataset. Despite MCP joint calcification seems to be a less specific indicator of CPPD compared to TFCC calcification, it can be helpful to differentiate CPPD from other conditions such as seronegative RA (11).

To the best of our knowledge, this is the inaugural CNN algorithm aimed at predicting CPPD via radiographic analysis. However, it is imperative to acknowledge that this algorithm alone is insufficient for diagnosing CPPD disease. Clinical and demographic parameters must also be considered to meet the ACR/EULAR criteria’s 54-point threshold (2). CPPD features in hand radiographs contribute to 16 points if a single joint is affected, 23 points if 2–3 joints are affected and 25 points if ≥4 joints are affected. As a next step, we are planning to add a model for the detection of OA in MCP-2 and -3, carpo-metacarpal-1 and scaphotrapeziotrapezoid joints. This would increase the ACR/EULAR criteria by further 7 points. Together with patient-reported information on age, joint distribution and comorbidity, this would be sufficient to make the classification for CPPD disease provided that entry criteria are met.

Ultrasound and dual-energy computed tomography play an important role in the diagnostic algorithm of CPPD (12, 13). Notwithstanding, radiography remains a crucial screening tool for CPPD due to its widespread availability and cost-effectiveness. Its ability to visualize the entire joint aids in assessing differential diagnoses or concurrent conditions. Furthermore, future machine learning pipelines may combine the identification of radiographic CPPD with other rheumatic conditions such as distal hand OA, RA, or psoriatic arthritis lesions (14, 15). Combined with further patient-reported outcomes and algorithms for predicting non-radiographic features, such as hand joint swelling from photographs (16), the scope of algorithms in remote patient monitoring could be expanded.

Machine learning models, like the one described here, extend beyond automation. Notably, heatmaps provide educational value by highlighting areas of interest, especially in positive classifications. Imaging remains the primary diagnostic tool in the absence of CPP crystals in the synovial fluid, requiring imaging of at least one symptomatic joint in patients not meeting sufficient criteria. This algorithm gains increased relevance in individuals with CPPD in four or more peripheral joints rather than monoarthritis. However, the diagnostic accuracy of radiography for CPPD and thus the algorithm’s veracity remains under-researched.

Limitations of this study include the dataset’s size, lack of external validation, and absence of data on different ethnicities. Hand radiographs with concomitant OA, RA, psoriatic arthritis or gout have not been excluded. The aim was to demonstrate the workflow of this algorithm and its interpretability for a basic classification task. Future tasks should aim to quantify CPP load, despite the current lack of evidence linking higher CPP loads to more severe CPPD disease. This could be explored in larger datasets using regression analysis similar to knee OA studies. The external validation should also be carried out in images from different X-ray devices, e.g., in “older” images with lower quality (higher noise, lower contrast-to-noise ratio).

Several avenues exist for improving or adapting our model. For instance, adjusting the threshold for our final model(s) could optimize its application, potentially incorporating different Fβ scores based on the relative clinical importance of precision and recall. Further accuracy enhancements could possibly be achieved by refining the input images, modifying preprocessing steps, or trialing different base models.

Overall, this work illustrates the feasibility and interpretability of using a deep-learning model to predict and detect CPPD in hand radiographs. Future improvements and validation efforts will involve larger and external datasets, accompanied by detailed clinical data analysis. Assessing user experience among radiologists and rheumatologists and addressing regulatory considerations and clinical workflow integration should be prioritized moving forward.

## Data Availability

The raw data supporting the conclusions of this article will be made available by the authors, without undue reservation.
