# Supplementary material for: Development of a deep learning model for automated detection of calcium pyrophosphate deposition in hand radiographs
Source: Front Med (Lausanne). 2024 Oct 23;11:1431333. doi: 10.3389/fmed.2024.1431333 (PMC11540928; doi:10.3389/fmed.2024.1431333)
Supplement: Supplementary file 1 [file Data_Sheet_1.docx]

SUPPLEMENTARY DATA

S1: Demographic data of CPPD+ or CPPD- patients in the different models.

|  | **Mean age** | **Female** | **Male** |
| --- | --- | --- | --- |
| **All** | 64.5 | 0.632 | 0.368 |
| **Combined+** | 75.0 | 0.665 | 0.335 |
| **Combined-** | 59.0 | 0.614 | 0.386 |
| **TFCC+** | 75.3 | 0.695 | 0.305 |
| **TFCC-** | 59.8 | 0.604 | 0.396 |
| **MCP2+** | 77.8 | 0.685 | 0.315 |
| **MCP2-** | 63.3 | 0.627 | 0.373 |
| **MCP3+** | 77.3 | 0.571 | 0.429 |
| **MCP3-** | 63.1 | 0.638 | 0.362 |

S2: Interobserver agreement per CPPD site with corresponding contingency tables

|  | Weighted kappa | Standard error | 95% confidence interval |
| --- | --- | --- | --- |
| TFCC | 0.91 | 0.01 | 0.88-0.94 |
| MCP 2 | 0.82 | 0.03 | 0.76-0.89 |
| MCP 3 | 0.90 | 0.02 | 0.85-0.95 |

TFCC

|  |  | Observer 2 | |  |
| --- | --- | --- | --- | --- |
|  |  | CPPD + | CPPD - |  |
| Observer 1 | CPPD + | 273 | 13 | 286 |
|  | CPPD - | 22 | 618 | 640 |
|  |  | 295 | 631 | 926 |

MCP 2

|  |  | Observer 2 | |  |
| --- | --- | --- | --- | --- |
|  |  | CPPD + | CPPD - |  |
| Observer 1 | CPPD + | 65 | 9 | 74 |
|  | CPPD - | 16 | 836 | 852 |
|  |  | 81 | 845 | 926 |

MCP 3

|  |  | Observer 2 | |  |
| --- | --- | --- | --- | --- |
|  |  | CPPD + | CPPD - |  |
| Observer 1 | CPPD + | 87 | 6 | 93 |
|  | CPPD - | 11 | 822 | 833 |
|  |  | 98 | 828 | 926 |
